# Granzymes—Their Role in Colorectal Cancer

**DOI:** 10.3390/ijms23095277

**Published:** 2022-05-09

**Authors:** Sara Pączek, Marta Łukaszewicz-Zając, Barbara Mroczko

**Affiliations:** 1 Department of Biochemical Diagnostics, Medical University in Bialystok, 15-269 Bialystok, Poland; marta.lukaszewicz-zajac@umb.edu.pl (M.Ł.-Z.); barbara.mroczko@umb.edu.pl (B.M.); 2 Department of Neurodegeneration Diagnostics, Medical University in Bialystok, 15-269 Bialystok, Poland

**Keywords:** granzymes, colorectal cancer, cancer, serin proteases, biomarkers

## Abstract

Colorectal cancer (CRC) is among the most common malignancies worldwide. CRC is considered a heterogeneous disease due to various clinical symptoms, biological behaviours, and a variety of mutations. A number of studies demonstrate that as many as 50% of CRC patients have distant metastases at the time of diagnosis. However, despite the fact that social and medical awareness of CRC has increased in recent years and screening programmes have expanded, there is still an urgent need to find new diagnostic tools for early detection of CRC. The effectiveness of the currently used classical tumour markers in CRC diagnostics is very limited. Therefore, new proteins that play an important role in the formation and progression of CRC are being sought. A number of recent studies show the potential significance of granzymes (GZMs) in carcinogenesis. These proteins are released by cytotoxic lymphocytes, which protect the body against viral infection as well specific signalling pathways that ultimately lead to cell death. Some studies suggest a link between GZMs, particularly the expression of Granzyme A, and inflammation. This paper summarises the role of GZMs in CRC pathogenesis through their involvement in the inflammatory process. Therefore, it seems that GZMs could become the focus of research into new CRC biomarkers.

## 1. Colorectal Cancer—An Urgent Need for Novel Biomarkers

Colorectal cancer (CRC) is the third most common malignancy in men and the second most common malignancy in women [1]. Notwithstanding the progress made in the detection and treatment of this type of cancer, CRC remains one of the leading causes of cancer-related deaths worldwide, with almost two million new cases each year [2,3]. 

Despite the fact that most many people may develop CRC, some risk factors have recently been identified and linked to tumour development. Those affecting cancer formation and progression may be divided into two main subgroups: environmental (modifiable) and genetic (non-modifiable) [4] (Table 1). It has been indicated that the interaction between environmental risk factors and genetic variations may contribute to increased CRC risk [2,3].

Depending on the origin of the mutation, CRCs are classified as sporadic, inherited, and familial [5,6]. Recent studies show that over 70% of CRCs are sporadic and occur in patients with neither a family history of CRC nor genetic predisposition. The process of CRC formation seems to lead to an accumulation of genetic changes that can cause the transformation from normal epithelium to adenomatous polyps, and ultimately to invasive colon cancer. Genomic profiling has demonstrated significant intra-tumour and inter-tumour heterogeneity, which is primarily due to the accumulation of a variety of genetic mutations and chromosomal aberrations during both disease initiation and progression [7]. It is assumed that there are three main mechanisms responsible for sporadic carcinogenesis in the colon [5,8]. They are chromosomal instability (CIN), microsatellite instability (MSI), and CpG island methylator phenotype (CIMP), which is a feature of the mutator phenotype.

The CIN mechanism has been found to be responsible for around 70% of sporadic CRCs. These tumours are mainly characterised by an accumulation of structural and numerical chromosomal abnormalities, which results in an aneuploid karyotype and chromosomal rearrangements [9]. This classical model of CRC development assumes the coexistence of several mutations in suppressor genes, such as APC regulator of WNT Signaling Pathway (*APC*) or tumour protein p53 (*TP53*), and overlapping mutations of proto-oncogenes, e.g., *K-RAS* (KRAS proto-oncogene), which activate the critical pathways that determine further progression [10].

The molecular mechanism of microsatellite instability is not fully understood. However, it is suggested that the MSI associated with carcinogenesis is due to the malfunction of proteins involved in DNA repair [11]. Damage to the DNA repair system may be responsible for the failure to remove the point mutations that arise during the preparation for cell division and with oncogenic factors during interphase. As a consequence, the number of mutations increase up to 700 times compared to cells with an efficient DNA repair system [12,13]. Analysis of selected microsatellite markers is important as it allows for the molecular classification of CRC, which takes into account such features as tumour location, tumour growth rate, cell malignancy, and the ability to metastasize. Moreover, an equally important issue concerns the molecular evaluation of selected microsatellite markers in inflammatory conditions, which increase the risk of the development of a neoplastic process. MSI occurs in hereditary and sporadic neoplasms, although with different frequency [13,14].

The third mechanism involved in the development of colorectal cancer is that associated with epigenetic instability, including DNA methylation. Then, many suppressor genes such as cyclin-dependent kinase inhibitor 2A (CDKN2A) are silenced by DNA methylation, which leads to disruption of cell division processes and apoptosis [15]. A group of neoplasms with a high level of methylation of many genes is known as the CpG Islet Methylator Phenotype (CIMP), which is diagnosed in approximately 15% of colorectal cancer cases. Thus, it is worth remembering that the mentioned mechanism leading to the development of colorectal cancer may overlap or occur at different stages of the course of carcinogenesis [16].

However, not all processes involved in colon carcinogenesis have been fully elucidated. It has been reported that most CRCs develop slowly, arising from benign lesions called polyps [17]. Initially, small polyps arise from large intestinal epithelial cells. Some of them grow excessively, develop dysplasia, and develop either into precancerous changes that take the form of benign adenomas or become cancerous [18].

Due to the high incidence and mortality rates of CRC, early detection is crucial to improving outcomes. This malignancy is commonly diagnosed at an advanced stage based on the following signs and symptoms: a change in bowel habits, cramping and abdominal pain, unintentional weight loss, rectal bleeding with bright red blood, blood in the stool, and fatigue [19]. However, the greatest challenge in CRC is its asymptomatic course since, particularly in the early stages of the disease, most patients do not report any alarming symptoms [20,21]. Currently, there are many commonly available tools used in the process of diagnosing CRC such as a comprehensive patient interview and a physical examination, as well as more specialistic tests including laboratory and radiological tests and histopathological examination [22].

**Figure 1:**
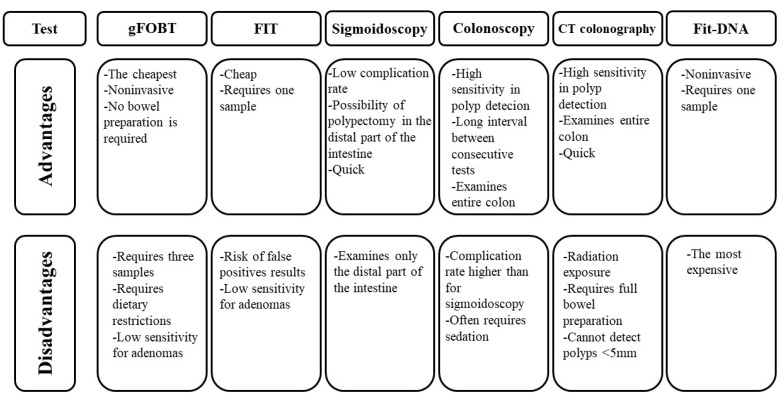
Advantages and disadvantages of selected screening tools in the diagnosis of CRC [24,25,26,27,28].

Regular CRC screening, which is recommended to begin at age 45 in the case of people at average risk of colorectal cancer, is crucial for the prevention and detection of this type of cancer. Screening for CRC (secondary prevention of CRC) involves the use of tests to detect cancer early, and thus reduce patient mortality [23]. It should be emphasized, however, that the effectiveness of screening for CRC is dependent not only on the use of a particular modality but also on the patient’s compliance. There are many colorectal cancer screening tests available, but each of them has its benefits and limitations. The characteristics of each test have an impact on the doctor’s decision regarding the selection of the most appropriate screening option for the patient. The most commonly used tests include stool tests: FOBT (faecal occult blood test), FIT (faecal immunochemical test) and FIT-DNA (also referred to as a stool DNA test), flexible sigmoidoscopy, colonoscopy, and computed colonography [24]. The advantages and disadvantages of the selected screening methods are presented on Figure 1 [25,26,27,28].

Despite the availability of multiple screening modalities, the search for new CRC biomarkers continues. A growing body of evidence suggests that ion channels are not only responsible for regulating homeostasis and ion potentials, but are also involved, inter alia, in cell proliferation and apoptosis [29]. Diseases that develop because of defects in ion channels caused by either genetic or acquired factors are called channelopathies. New studies show that ion channels and transporters (ICTs) are one of the factors involved in carcinogenesis, and their abnormal expression or activity may contribute to the malignant transformation as well as progression of many gastrointestinal cancers, including CRC [30]. The best-characterised channels are potassium, chloride, calcium, sodium, and zinc. Most of these channels act as oncogenes in the pathogenesis of CRC (KCNC1, KCNN4, CLIC1, TRPC1, SCN1A, ZnT5) and some of them can suppress tumour growth (CLCA1, CLCA2, TRPM6, stim2, SCN8A) [29,31,32].

## 2. Granzymes—Structure, Function and Apoptosis

T lymphocytes (T cells) and Natural Killer (NK) cells are specialised to track down cancerous or virus-infected cells in the human body. The trait that defines cytotoxic lymphocytes relates to the expression and release of potent toxins [33]. They produce the so-called granzymes—special enzymes of the immune system that are capable of causing the self-destruction of infected cells [34]. Granule-associated enzymes (GZMs) are members of the serine proteases family and are structurally related to chymotrypsin. They have highly conserved residues at positions 1–4 and 9–16, and they also commonly have three conserved disulphide bridges. The classification of these proteins includes their cleavage specificity. Five human GZMs have been found: Granzyme A (GZMA), GZMB, GZMH, GZMK, and GZMM [35], while seven GZMs have been discovered in rats (GZMA-C, GzmI-K, and GzmM) and ten in mice (GZMA-G, GZMK-M) [36,37]. Although human GZMs show high homology (40%) in the amino acid sequence, they differ in the specificity of their primary substrate—the amino acid that is cleaved most preferably by GZMs—which leads to their specific degradation [38].

GZMs are a key element in the pathogenesis of many diseases, contributing to the progression of cardiovascular diseases, diabetes, atopic dermatitis, and sepsis [39,40,41]. Studies have demonstrated that inhibiting GZMB reduces disease severity in autoimmune blistering diseases. Thus, enhanced GZMs expression is not protective. In fact, GZMs are involved in a number of pathologies, suggesting that their cytotoxic activity requires regulation of both the production and release of GZMs. This is crucial for the effective and, principally, safe functioning of cytotoxic immune cells [42].

It is commonly known that selected GZMs show unique substrate repertoires and can act both intracellularly and extracellularly. The extracellular presence of granzymes may be the result of immune synapse leakage during cytotoxic T lymphocyte responses. Alternatively, another theory suggests that GZMs could be actively secreted during inflammation [43]. As for the remaining serine proteases, the catalytic activity of GZMs is dependent on a serine residue at the active site. Although the ability of GZMs to eliminate target cells by various mechanisms and types of induced cell death is still considered their major function, it has become clear that not all granzymes have specific cytotoxic functions. These proteins, found in extracellular human fluids, contribute to the inflammatory response and processes related to the degradation of the extracellular matrix (ECM), as well as vascular permeability and dysfunction, the release of matrix-sequestering growth factors, or receptor activation [44,45]. However, elimination of specific target cells seems to be the key aspect of the efficacy of cancer immunotherapy. Targeting the cytotoxic effects of T lymphocytes appears to be important for improving treatment efficacy without enhancing side effects [46].

For a number of years, scientists have tried to determine the mechanism by which GZMs can penetrate inside infected cells, and two potential models have been proposed [47]. In the first model, GZMs (GZMB) are transported across the cell membrane after attachment to cell surface heparan sulphate (HS). Although the membrane transport theory has a solid foundation, it has been observed that HS binding to GZMB causes side effects [48]. Hence, a suggestion that an alternative mechanism is necessary for the application of GZMB-based therapies in humans has been put forward.

**Figure 2:**
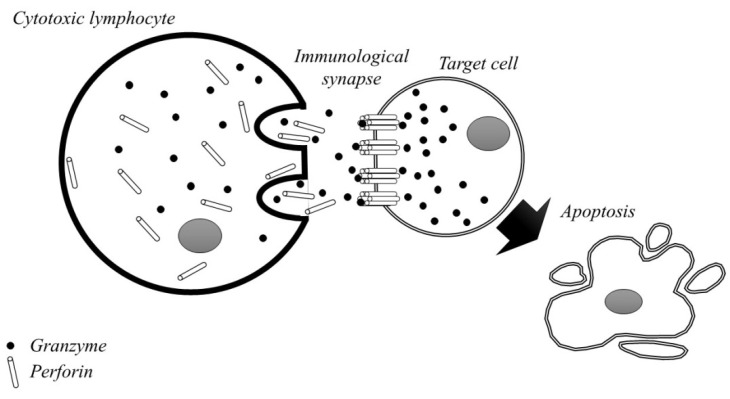
The formation of the granzyme/perforin immunological synapse.

In the second model, GZMB enters the target cell through unique channels in the disrupted lipid membrane. Cytotoxic cellular apoptosis is induced via the granular secretion or the death receptor pathway [49]. After recognising the target cell, cytotoxic cells are able to start releasing the content of certain granules into the immune synapse. Perforin provides GZMs with access to the cytosol of the target cell, where GZMs cleave their complex with substrates and begin to promote programmed cell death [50,51,52]. Since the pores made of perforin are very small and only permeable for a short time, scientists have favoured the membrane transport model. However, membrane-transported GZMs can also damage healthy cells. The creation of the perforin/granzyme tuner is shown in Figure 2 [53]. Apoptosis is one of the natural biological processes of programmed and controlled cell death in the body [54]. This mechanism is necessary and has a positive effect on proper development and homeostasis, and it also prevents excessive proliferation of harmful cells in the body. Used, damaged, or unnecessary cells are constantly removed, and new ones are created in their place [55]. Initiation of apoptosis can occur extrinsically or intrinsically and it leads to a number of biochemical and morphological changes in the cell. During this process, the cell shrinks and the cell membrane is bubbled. Nuclear DNA fragmentation and nucleus condensation also occur. As a result, the integrity of the cell membrane in the early phase of apoptosis is preserved. Then, apoptotic cells are removed by phagocytes before they become lytic [56,57,58]. This process is critical to avoid inflammation or autoimmunity, which are undesirable phenomena in the body. Since apoptosis cannot be stopped or reversed, there must be effective mechanisms to regulate this process. Caspases and Fas receptors stimulate the process of apoptosis, while Bcl-2 proteins have an inhibitory effect. Although Granzyme A and Granzyme B represent different apoptotic pathways, they share a common mechanism for reaching the target cell through the pores in the cell membrane [59,60].

GZMB can induce two types of apoptotic cell death: intracellular apoptosis, which is perforin-dependent, and extracellular perforin-independent apoptosis, called anoikis. Anoikis is a mechanism activated in certain cell types by the loss of connectivity with the matrix or with other cells, resulting in apoptotic cell death. It plays a role in preventing inappropriate translocation of cells and allows for the elimination of detached cells, thus maintaining tissue homeostasis [61,62,63]. On the other hand, GZMA activates the caspase-independent cell death pathway with morphological features of apoptosis. Nevertheless, it has unique substrates and mediators. After entering the cytosol of target cells, GZMA is transported into the mitochondria via the translocase of the inner membrane (Tim)/translocase of the outer membrane (Tom)/protein-associated motor (Pam) import pathway [64].

## 3. GZMs in Inflammation

Inflammation is a multi-step process that is a physiological response to infection or tissue damage. It is essential for survival and has a proven beneficial effect on the neutralisation of any dangerous or harmful factors [65,66]. This process is closely related to the various anti-inflammatory mechanisms that maintain tissue homeostasis in the body. Certain circumstances disrupt immune homeostasis, which can lead to acute and chronic inflammatory states, including cardiovascular, pulmonary, metabolic diseases, and even cancer [67,68,69,70]. Understanding the mechanisms involved in both the development and progression of pathological states is crucial to finding effective therapies. Although the function of GZMs as immune regulators was suggested decades ago, they are still of interest due to discoveries in which certain GZMs were shown to have the influence on stimulating cytokine expression, thereby promoting inflammation [71,72]. As already mentioned, it is now known that individual GZMs may act both intracellularly and extracellularly and may contribute to increased vascular permeability and disfunction. Growing evidence demonstrates that extracellular GZMs are strongly involved in the modulation of inflammatory pathways [73] since increased GZMs concentrations have been observed in the sera or plasma of patients suffering from inflammatory diseases such as rheumatoid arthritis (GZMA, B), viral infections (GZMA, B, K), sepsis (GZMA, B, K, M), and acute airway inflammation (GZMK) [74,75,76]. A recent study found that GZMM is also involved in the early stages of mucositis as GZMM knockout mice exhibit increased inflammation in a mouse model of ulcerative colitis. The above observations prompted researchers to investigate the other functions of these proteases in inflammatory processes. Consequently, it was proven that particularly GZMA and GZMK, by acting as extracellular proteases, regulate the inflammatory response independent of their ability to induce cell death [77]. However, the molecular mechanisms by which GZMs directly release pro-inflammatory cytokines still remain unclear. While GZMK is able to cleave and activate Protease Activating Receptor 1 (PAR1), thereby leading to the release of cytokines from fibroblasts, GZMA can convert pro-IL-1β into bioactive IL-1β in human monocytes [77,78]. In addition, GZMA is able to mediate the release of proinflammatory cytokines from several cell types such as monocytes, epithelial cells, and fibroblasts. It has also been observed that GZMs may interfere with the LPS-TLR4-induced cytokine response during antimicrobial innate immune responses [79].

## 4. Inflammation and Carcinogenesis

Inflammation is a predisposing factor to cancer development and plays a role in promoting all stages of tumour formation. Cancer cells interact with the surrounding stromal cells and inflammatory cells to create an inflammatory tumour microenvironment (TME) [80]. The link between inflammation and neoplasms has been originally suggested based on the observation of inflammatory cells like macrophages in tumour biopsies [81,82]. Macrophages, neutrophils, fibroblasts, and epithelial cells interact through the release of multiple mediators that support both inflammation and carcinogenesis [83,84,85,86,87,88]. However, little is known about the molecular mechanisms that control the production of these pro-inflammatory factors during both gut inflammation and CRC. Therefore, the regulation of inflammation by pharmaceuticals is not entirely specific and is associated with serious side effects in many cases [89,90].

Chronic inflammation is one of the unfavourable prognostic factors for the progression of solid tumours such as CRC [91]. It is well known that patients with ulcerative colitis (UC) are at increased risk of developing CRC [92]. Moreover, it has also been demonstrated that inflammation is involved in both sporadic and hereditary CRC [93]. Pathological analysis of CRC tissues has revealed infiltration of various cell types such as neutrophils, mast cells, NK cells, dendritic cells (DCs), and tumour associated macrophages (TAMs) [94]. An important element is also the participation of these cells in the recruitment and interaction with other cells involved in various types of immune responses, which in turn leads to a balance of immune surveillance. This phenomenon helps in the early detection of abnormal foci and allows for the elimination of potentially abnormal cells [95]. However, it is important to remember that chronic inflammation creates a favourable microenvironment that overwhelms immune surveillance. Some researchers have suggested three main patterns by which inflammation could be linked to CRC. The first of them—inflammation-associated tumorigenesis—demonstrates that chronic inflammation may result from infection or dysregulated immune response, thus promoting tumorigenesis via, e.g., DNA damage. The second is when tumour-elicited inflammation initiates the inflammatory response, and the third is when therapy-induced inflammation may trigger inflammation that promotes tumour development by releasing damage-related molecular patterns (DAMP) from necrotic cells [96,97,98].

As inflammation is one of the major risk factors for CRC, it has been suggested that GZMs, which have pro-inflammatory properties, are involved in the development and progression of many cancers, including CRC. A better understanding of the mechanisms involved in the development of CRC may lead to the identification of new targets as well as improvement in current anti-CRC therapy [99,100].

## 5. GZMs in CRC

The immune system has evolved a number of mechanisms that are aimed at protecting the host organism against pathogens, as well as cancer, while maintaining self-tolerance, which is prevents the body from having an autoimmune reaction [86]. Among these mechanisms, both NK and Tc cells are able to recognize and kill target cells. However, an important element in immune response is control mechanisms, a possible lack of which contributes to the development of the pathology observed, among others, in inflammatory or autoimmune diseases. This may contribute to both tumour initiation and progression [101]. NK and Tc cells use, inter alia, the granular exocytosis pathway, which occurs through the coordinated action of perforins, and GZMs. Previously, GZMs were associated with activating biological functions by inducing cell death. However, research performed over the past few years and the fact that the expression of GZMs has also been demonstrated on cells other than NK and Tc indicates that some GZMs, particularly GZMB, may play key regulatory functions in the development and progression of neoplasms by participating in inflammation, angiogenesis, and immune homeostasis [102].

A number of investigations conducted in recent years have revealed that both human and murine GZMs, such as GZMA, GZMM, or GZMK, are also involved in various processes not related to cytotoxicity—for example, in regulating the inflammatory response [71]. Some evidence indicates the influence of selected GZMs on carcinogenesis and their role in CRC pathogenesis. The association between GZMs and malignant diseases has been suggested since elevated levels of circulating extracellular GZMA and GZMB have been related to several inflammatory diseases [72].

GZMA is a member of the serine proteases family, which is commonly recognised as anti-tumour and anti-infective agents because of its ability to trigger cell death on target cells in vitro [103,104]. Nevertheless, the direct relationship between this granzyme and colon carcinogenesis remains unclear. A study by Santiago et al. [105] assessed the importance of GZMA in promoting CRC. Transcriptome analysis showed a strong correlation between the expression of GZMA and inflammatory genes found in most CRC molecular subtypes. Moreover, it was found that GZMA deficiency and its therapeutic inhibition have an effect on reducing inflammation as well as intestinal permeability. These results may suggest that GZMA, which acts in the extracellular environment, is responsible for regulating the inflammatory response and might be a key mediator in the development and progression of CRC [105]. Additionally, the authors revealed that extracellular active GZMA induces IL-6 expression in M1 macrophages. High infiltration of macrophages has been correlated with improved survival among CRC patients. Thus, all these results confirm that GZMA exerts its pro-inflammatory carcinogenic function from the extracellular space, which is a key finding in designing therapeutic approaches to block GZMA activity [105]. These observations may indicate that GZMA might participate in a novel mechanism contributing to the development of CRC in vivo by regulating inflammatory responses. However, it has been found that therapeutic inhibition of GZMA extracellular activity reduces inflammation as well as the incidence of cancer. This makes GZMA the most viable therapeutic option for preventing or treating cancers, particularly those in which inflammation plays an important role, such as CRC. Inhibition of GZMA seems to prevail over conventional cytokine-targeted methods as blocking pro-inflammatory cytokines, such as TNF-alpha and IL-6, may increase patients’ susceptibility to bacterial, viral, and fungal infections [60].

GZMB is a pro-apoptotic cytotoxin that has the strongest effect on the granular exocytosis pathway of cytotoxic lymphocytes [106]. This protein is localised inside endosomes and is synthetised as an inactive precursor called zymogen (proGZMB) [106]. It is activated via cathepsin C (CatC), which is responsible for removing the Gly-Glu N-terminal dipeptide. In fact, residual GZMB activity has also been observed in mice lacking catepsin C, which suggests that other zymogen convertases are also present [107].

GZMB is produced and secreted both by immune cells such as monocytes/macrophages, B and T cell subpopulations, basophils, mast cells, and non-immune cells such as keratinocytes, pneumocytes, chondrocytes, or smooth muscle cells [108]. Moreover, the expression of GZMB has also been demonstrated on neoplastic cells, e.g., cancer of the breast, prostate, pancreas, epithelium of the urinary tract, and colon [41,109,110,111]. A study by Pages et al. [112] demonstrated increased GZMB expression in relation to normal mucosa of the colon. Similar dependences were observed by Salama et al. [113], who revealed that GZMB expression is higher in tumours with microsatellite instability, dense lymphocyte infiltration, and proximal colon location, but lower in tumours with, inter alia, vascular invasion. The authors observed that increased expression of GZMB was associated with improved survival among CRC patients [113]. One of the most important findings of a study by Salama et al. [113] was decreased expression of GZMB in patients with pathological metastases. Referring to the work of Mulder et al. [114], a reversal of the relationship between GZMB expression and tumour stage was also observed. The above results suggest the presence of a special immunological activity of the anti-tumour response, which affects patient outcomes.

GZMM belongs to a group of serine proteases that are often expressed in NK cells and, in combination with perforin, induce apoptosis of target cells. Due to the fragmentary information and incomplete understanding of how it functions, GZMM is sporadically referred to as one of the ‘orphan granzymes’ [115]. However, due to a lack of a clear consensus regarding both the substrates and the pathways involved in in vitro processes, the cytotoxic potential of this molecule is highly controversial. Recent studies suggest a protumoural role for GZMM in EMT. GZMM expression in human tumour tissue has been shown to correlate with cells expressing the EMT phenotype. In addition, tumour cell lines in which GZMM was downregulated induced less distant metastases in murine models [115]. However, the mechanisms behind the above process are not fully understood. The authors suggest the activation of STAT3 signalling, but it is still unclear how this pathway could be activated in the culture of a CRC line where cytokines such as IL-6 should not be present. Although the results in the mouse model have recently been questioned [116] the findings in humans might be potentially interesting. However, further experimental evidence is needed to elucidate the role of GZMM in the development and/or progression of CRC. The role of selected GZMs in CRC is presented in Table 2.

## 6. Conclusions

Due to global environmental and demographic changes, an increasing trend in the incidence of CRC has been observed. Reducing CRC incidence and mortality rates is a major challenge for the healthcare systems worldwide. A novel way to understand the biological processes involved in CRC will have a profound impact on future diagnosis and treatment of patients. Hence, the growing incidence of colorectal cancer requires the dynamic development of innovative methods and diagnostic tests that would allow for early cancer detection. As the understanding of the importance of GZMs continues to evolve, it has become clear that both the physiological and pathological roles of these enzymes are far more complex than previously thought. Several studies suggest that GZMs play an important role in the pathogenesis of CRC. The ambiguous relationship between these molecules and malignancies is intriguing, and the results of studies on the expression of these proteins in CRC are very promising. GZMs, particularly GZMA, are able to actively participate in the release of inflammatory mediators including IL-6. Thus, they contribute to the transformation of epithelial cells and tumour progression. Furthermore, a deficiency of GZMA was found to be associated with both reduced gut inflammation and arrested CRC development, which may find application as a therapeutic target. On the other hand, GZMs may have anti-inflammatory properties and their increased levels might be associated with a good prognosis. Low expression of GZMB has been related to the presence of metastases, which suggests its anti-tumour activity. In conclusion, GZMs presented in this review paper may hold promise for applications as potential diagnostic and prognostic factors of CRC.

## Figures and Tables

**Table 1:** Modifiable and non-modifiable risk factors of CRC.

Non-Modifiable Risk Factors	Modifiable Risk Factors
age sex ethnicity family history personal history of adenomas polyposis syndromes inflammatory bowel disease BRCA gene mutations	red meat consumption processed meat consumption smoking tobacco alcohol abuse low-fibre diet overweight and obesity lack of physical activity

**Table 2:** The role of selected GZMs in CRC.

Protein	Type of Expression	Method of Detection	Effect	Correlation with Inflammatory Genes	Suggested Role	Ref.
Granzyme A	mRNA expression	RT-PCR	- significantly elevated expression was observed in CRC and inflammatory samples in comparison to control group	yes	- promotion of tumour development - progression of CRC	[105]
Granzyme B	mRNA expression	RT-PCR	- increased levels were associated with absence of pathological signs of early metastasis invasion	nd	- antitumour activity	[114]
	protein expression	IHC	- low expression was associated with early signs of metastasis - high expression was associated with better survival	nd	- prognostic factor	[113]
	mRNA expression	RT-PCR	nd	no	nd	[114]

## Data Availability

Not applicable.
